# Research priority-setting: reproductive health in the occupied Palestinian territory

**DOI:** 10.1186/s12978-018-0472-0

**Published:** 2018-02-13

**Authors:** Niveen M. E. Abu-Rmeileh, Rula Ghandour, Marina Tucktuck, Mohammad Obiedallah

**Affiliations:** Institute of Community and Public Health, Birzeit University, P.O.Box 14, Birzeit, West Bank, oPt Palestine

## Abstract

**Background:**

Occupied Palestinian territory (oPt) is an authority with limited resources. Therefore, research conducted in such a setting should be prioritized and coordinated to follow a national research agenda. This study aims to produce a research agenda for reproductive health in the oPt that can be utilized by reproductive health stakeholders and contribute to the development of policy-based evidence to guide health practice.

**Methods:**

In the current study, we followed research prioritization methods developed by the World Health Organization-Child Health and Nutrition Research Initiative. Research questions were obtained from reproductive health experts in the oPt. The questions were then grouped into thematic areas which were prioritized by the reproductive health experts. Scores were calculated and sorted to define the top priority research areas.

**Results:**

A total of 232 research questions were prioritized by 30 reproductive health experts. Health system issues were the most addressed in the top 50 research questions. They included questions on the quality of services and health professionals’ knowledge and continuous professional training. Adolescents’ sexual and reproductive health and gender-based violence were rarely mentioned in the top 50 questions. The number of questions related to safe motherhood was around 50% followed by questions related to health system. Questions related to elderly women and menopause as well as reproductive system cancers were also within the top 50 ranked questions.

**Conclusions:**

Priority research areas in reproductive health were identified for the oPt, which should be utilized by researchers with a focus on the high priority areas. Policy makers and funders should coordinate their efforts to ensure the production of research with value to the Palestinian context, in the most efficient way possible.

## Key messages

- Reproductive health experts in the occupied Palestinian territory were able to identify previously unaddressed priority research areas related to reproductive health
- Future research needs to be guided by the research priority list to maximize efficient utilization of resources
- The quality of services within the health system were among the top priority research areas identified, whereas questions related to adolescents sexual and reproductive health were least prevalent

## Plain English summary

Policy makers today are more reliant upon evidence-based policy planning than ever before, which increases the demand for research production. To keep up with this increasing demand, it is especially important for countries with limited resources, such as the occupied Palestinian territory (oPt), to identify priority research areas in order to focus their resources on the needed evidence and reduce duplication of existing studies. This study aims to identify a list of the most important reproductive health research questions proposed by Palestinian health providers and experts. The participants included most of the stakeholders and experts in reproductive health service provision and research in the oPt. These experts identified research questions which were grouped by the researchers into common themes. These themes were further prioritized by the experts based on standardized methods. A list of 50 research questions were identified. Several questions related to the health system, including quality of care, health professionals’ knowledge and preparedness, access to and availability of services, were among the most important questions. Questions related to high-risk pregnancies and maternal mortality were also within the 50 top research questions. Few questions on adolescents’ sexual and reproductive health and gender-based violence were within the top 50 research questions. Future research should focus on the identified high priority areas. Policy makers and funders should work in coordination to ensure the production of research with value to the Palestinian context.

## Background

Around 85% of research investment worldwide is wasted as reported by Chalmers et al. A waste in research was considered, “when the need of potential user of research evidence was ignored or when available evidence was overlooked” [1]. The reasons for waste included choosing the wrong research question, conducting studies that are poorly designed, and failing to publish unusable sections of research [1]. It thus becomes imperative to set research priorities to help maximize evidence utilization and reduce research waste, especially in low-income countries with limited research resources [2].

In the occupied Palestinian territory (oPt), research is mainly conducted by academic institutions, occasionally in collaboration with health providers. Research conducted is mainly selected based on consultation with health providers, observation, and experience of the researcher in a specific field or in response to a funder and/or health provider request [3]. The number of published papers focusing on health in Palestine/oPt has increased since 2010 compared to previous years. However, only 29% of these papers were cited more than 5 times [4], which might indicate local rather than international importance. In addition, it might indicate that these studies are not addressing the right questions.

In relation to reproductive health (RH) research, a comprehensive review on maternal and child health in the oPt described the situation with respect to the fourth and fifth Millennium Development Goals on reducing child mortality and improving maternal health, respectively. The review also identified several research areas as well as reported the type of research conducted and the quality of available data [5].

Several research priority exercises were conducted to address the global, and more specifically, low and middle-income countries needs [6–9]. However, a country-specific research priority list is imperative to help provide the needed evidence for program and intervention planning at the national level. Therefore, this study aims to build a research agenda for RH in the oPt that can be utilized by RH stakeholders and contribute to the development of policy-based evidence to guide health practice.

## Methods

We used the reproductive health definition adopted by the International Conference on Population Development (ICPD) Program of Action held in Cairo in 1994. In the current study, we followed research prioritization methods developed by the World Health Organization’s (WHO) Child Health and Nutrition Research Initiative (CHNRI) [10]. The prioritization exercise was implemented in three phases: (1) the generation and collection of research questions, (2) consolidation of research questions and thematic analysis, and (3) the prioritization exercise of the research questions using pre-defined scoring criteria.

### Phase 1

The main stakeholders in RH in the West Bank and Gaza Strip were identified in part by consulting the stakeholders who participated in the Palestinian National Reproductive Health Strategy and Action Plan 2014–2016. These stakeholders mainly included the Palestinian Ministry of Health and other governmental ministries, the United Nations Relief and Works Agency for Palestine Refugees (UNRWA) and national non-governmental organizations as well as a number of private physicians. In addition, researchers representing Palestinian academic institutions were included, thus tapping into the clinical aspect of reproductive health alongside the academia aspect. Invitations were sent to the identified participants of 45 individuals from 21 national organizations, through email, fax, and phone. Out of those who were approached, 34 individuals from 19 organizations responded positively to the invitation (individual response rate of 75.6%). Participants were asked to propose six research questions in the field of reproductive health; each based on his/her expertise, knowledge and experience. Phase 1 was completed between December 2015 and January 2016.

### Phase 2

The list of proposed research questions was independently reviewed by two researchers. A third reviewer evaluated the list of questions from the two reviewers and resolved discrepancies. A reduced and refined list of research questions was prepared for thematic analysis. Phase 2 was completed between January 28th – February 18th, 2016.

### Phase 3

A total of 32 individuals from 19 organizations were invited to participate in the research priority scoring exercise, through email, phone, and fax (where appropriate). Of the total invited participants (who also participated in phase 1), 30 individuals from 16 organizations responded positively to the invitation (individual response rate of 93.8%) and four responses were received by email because of mobility issues. Phase 3 was completed between February 28th – April 1st, 2016.

The scoring criteria adopted was based on the WHO-CHNRI guidelines [11] (Table 1).

**Table 1:** Scoring criteria for the research priority-setting exercise in reproductive health

Criteria^a^	Definition	Score and explanation
1. Answerability	The research question can be ethically answered	0- Cannot be answered 5- Can be fully answered 0–1–2-3-4-5
2. Effectiveness	The new knowledge is likely to result in an effective intervention or program in the reproductive health field	0- Not effective 5- Very effective 0–1–2-3-4-5
3. Deliverability	The research is likely to generate new knowledge that can help in improving reproductive health in an acceptable and affordable manner	0- Cannot be delivered 5- Can be fully delivered 0–1–2-3-4-5
4. Potential impact	The results of this research will have a public health impact (improve the reproductive health from the public health perspective)	0- No Public health impact 5- High public health impact 0–1–2-3-4-5
5. Equity	The research will include and target the most vulnerable sectors of society	0- Not equitable 5- Equitable 0–1–2-3-4-5

^a^Criteria follow the World Health Organization’s Child Health and Nutrition Research Initiative (WHO-CHNRI)

An excel spreadsheet with the research questions was shared with the participants. They were then instructed to score each research question against the five criteria, where each criterion had a score range of 0–5. In addition, in the case that participants were not able to make a judgment on a research question, they had the option of indicating ‘I don’t know’ and ‘not applicable’ on the spreadsheet.

#### Data analysis

The metrics-based approach (pooling individual rankings), as employed by the WHO-CHNRI, was used for data analysis. For each research question, an unweighted mean score was calculated by summing up the individual scores of each criterion. No special weighting was applied to the criteria. ‘I don’t know’ and ‘not applicable’ was treated as missing and was not included in the calculation of the average. The resulting score of each research question was obtained and the research questions were ranked and prioritized according to their score. The top 50-research questions with a total score of 85+ were reported in the results section.

## Results

**Fig. 1:**
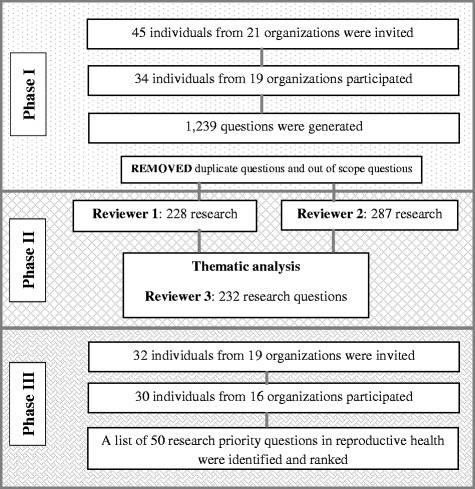
Study and analysis flow by phases

Figure 1 shows the study and analysis flow of the three phases. A total of 34 participants from 19 organizations responded positively to phase 1 invitation and provided 1239 research questions in reproductive health. After removing duplicate and out of scope questions and conducting thematic analysis, a total of 232 research questions emerged covering three main thematic areas (health systems, community-related and individual-related aspects within reproductive health). Out of the 34 participants, 30 individuals from 16 organizations partook in phase 3 scoring exercise (individual response rate of 93.8%). A list of 50 research questions was identified as priority research areas in reproductive health in the oPt.

### Priority areas

**Fig. 2:**
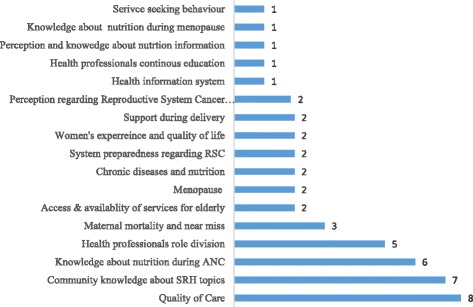
Distribution of the top 50 research questions divided by main areas

Figure 2 presents the distribution of the top 50 research questions divided by main reproductive health areas. Eight questions out of the 50 were related to the quality of care, six were related to knowledge of health professionals about nutrition during antenatal care period, and five were related to health professionals’ role division (tasks) and responsibilities. These three areas, in addition to others listed in the figure, accounted for almost 60% of the top 50 priorities, and fall under health system. Questions related to community knowledge about reproductive health areas, including school sexual and reproductive Health (SRH) programs, preconception and delivery, accounted for 15% of the top 50 questions. Moreover, the top 50 priorities included new topics such as menopause, chronic diseases and knowledge about nutrition at different stages of the reproductive life. Important topics such as reproductive system cancers were also in the list with one or two questions focusing on community and women’s knowledge, preparedness and coping with cancer diseases.

### Reproductive health topics

**Fig. 3:**
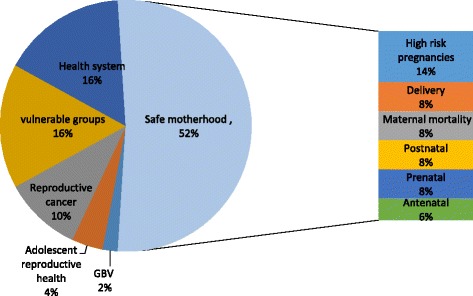
The top research priority areas divided based on ICPD components

An in-depth look at the top 50 questions with the highest score following ICPD 1994 components, we found that 52% of the research questions were about safe motherhood. Within safe motherhood priority area, high-risk pregnancy received the highest number of research questions while preconception, antenatal, delivery, postnatal and maternal mortality received almost a similar number of research questions. Health system and menopause management related questions each accounted for 16% of the top 50 questions. There was only one research question on gender-based violence and two questions on adolescent reproductive health issues (Fig. 3).

### Priorities by respondents

Finally, research priority areas varied among respondents. For instance, health service providers raised questions about community knowledge, attitude, and perceptions towards reproductive health services. In addition, they raised questions on specific topics, such as reproductive system cancers, abortion (and unwanted pregnancies) services and reproductive tract infections (RTIs) and sexually transmitted infections (STIs). The clinical gynecologists raised questions about the availability and affordability of services, mainly pre-conception, infertility, abortion and miscarriages, postnatal and family planning services, and the availability of related protocols. Moreover, academia raised questions about health professionals’ preparedness to work with adolescents, support for women during prenatal, delivery and postnatal, in addition to the availability, affordability, and quality of reproductive health services (Table 2).

**Table 2:** Top (highest scoring) research priority questions by reproductive health experts

Service Providers
Assess community preparedness and acceptability regarding reproductive system cancers
Evaluate the level of community knowledge, attitudes, and perceptions towards prenatal, antenatal, and postnatal services; RTI/STIs; abortion and miscarriage services; family planning and contraception; and the role of men in family planning
Evaluate the level of community knowledge, attitudes, and perceptions towards reproductive system cancers including screening and detection
Academia
Assess and evaluate the support system for women with reproductive system cancers during labor and the postnatal period
Assess and evaluate the methods adopted by health professionals in spreading awareness and education on reproductive health among adolescents
Understand the Epidemiology (prevalence/ distribution/ determinants/ associated factors) of high risk pregnancies
Evaluate the effectiveness of existing reproductive health services to prevent post-partum hemorrhage
Assess the availability of the types of services provided for family planning, postnatal care, labor, and delivery
Clinical Gynecologist
Assess the availability of the types of services provided for antenatal care, reproductive system cancers, nutrition, and infertility
Assess the protocols and guidelines implementation cycle (availability, comprehensiveness, knowledge, training, and application) regarding early marriage and teenage pregnancy
Assess the affordability and access to Antenatal care, postnatal care, labor, abortion, and miscarriage services
Other Ministries
Assess the availability of the types of services provided for antenatal care; reproductive system cancers; RTIs/STIs; menopause; infertility; GBV; early marriage and teenage pregnancies
Understand the Epidemiology (prevalence/ distribution/ determinants/ associated factors) for post-partum hemorrhage

## Discussion

The current study identified the national research priority areas within reproductive health in the oPt. The list is comparable to the one identified for low and middle income countries by de Francisco et al. [12]. A wide range of questions were raised, including simple epidemiology questions, health system, and social and behavioral questions. However, the list lacked specific clinical, operational, or intervention questions. As for reproductive health topics, questions related to safe motherhood were more than 50% of the questions followed by health system related questions. Adolescents health and gender-based violence questions were least mentioned. Interestingly, a greater emphasis was given to questions related to elderly women in their menopausal phase. Usually, this group is categorized within the marginalized or vulnerable groups. It is also worth mentioning that topics such as abortion and sexually transmitted infections (STI) were neither in the top 20 nor 50 priority areas. Although they were mentioned by different local experts, they did not reach enough consensuses to be ranked highly.

The process of setting research priorities in low and middle-income countries is challenging since it requires the involvement of all stakeholders to come to a national consensus. In our study, Palestinian experts and stakeholders working in reproductive health at the governmental and non-governmental sectors, universities, gynecologists, and statistical offices participated in this exercise. The challenge we faced was at the beginning of this exercise because some stakeholders did not understand the process and aim of the research priority setting and they thought it is funding priorities for reproductive health services. However, once they understood the aim and importance of the exercise, they became motivated and had almost a hundred percent response rate. This is in line with Rudan’s et al. main recommendation to improve the process of prioritization in health research by increasing the acceptability and popularity of such process with local policy makers [13].

The results of this study provide local and context-specific questions which cannot be compared to other countries specifically; nevertheless, they are in line with what has been reported by de Francisco et al. [12]. The results were general and not specific as reported in other studies since our study asked for general research questions while the other studies focused on interventions [7, 14] or specific governmental services [15]. In fact, research priority setting is an iterative process, and this is only the first step in a much longer process of refining the research priority areas into more specific questions. Hence this list should be reviewed and updated regularly. Based on the presented RPS findings, area specific research priority setting should be conducted for some topics, such as family planning and adolescents health, where validated methods were developed and used previously [6, 7, 16]. These areas were mentioned by different experts but did not reach consensus on specific questions.

The priorities were not similar across different respondents, perhaps because each was gearing the question toward their interest and experience. For example, the gynecologists were asking about unwanted pregnancies and abortion, health providers were interested in how to address cultural and norms barriers to family planning services, and other ministries were inquiring on how to improve family planning awareness and education, etc. Service providers in general were interested in understanding factors that are beyond their control, including cultural norms and beliefs, to improve their services. As for academics, in addition to understanding the quality of available health services, they were also interested in understanding the burden of selected topics locally that is of international importance. Although variation exists, the overall responses were complementary, and a comprehensive list of research priority was produced and agreed upon by all respondents.

### Strengths and limitations

This study provides the first evidence-based research priority setting in reproductive health in the oPt. The output is a list that highlights the main areas that need to be researched to inform health policy. A slight modification to priority setting process was applied in the last phase where we invited the different stakeholders to participate in a workshop for the final scoring exercise rather than continue the communication through e-mail. This step was taken to ensure a high response rate and to receive the responses in a timely fashion. We had a high response rate at the different data collection levels which emphasizes the agreement of all experts in the reproductive health field on having national research priorities for reproductive health. We acknowledge the limitation that the study excluded neonatal health from the priority setting as these topics were not mentioned by the experts in phase 1.

Given that priority setting in health research is not a theoretical exercise with a single possible correct outcome and final decisions depend on the context [10], various approaches exist to guide the process of research priority setting [17–20]. In this study, we tried to maintain a high quality prioritization process following the CHNRI method which is a standardized method that has been used internationally and in different countries at the national level [2].

## Conclusions

Priority research areas in reproductive health were identified. The research priority list should be utilized by researchers, policy makers and funders to ensure that the results of the conducted research are of value to the Palestinian population and context.
